# Ultrasound-assisted H_2_O_2_ degradation enhances the bioactivity of Schizophyllan for wound healing and tissue regeneration

**DOI:** 10.3389/fphar.2025.1562839

**Published:** 2025-03-20

**Authors:** Hui He, Yu Liu, Qingpeng Li, Fenrou Chen, Lin Zhou

**Affiliations:** ^1^ School of Life Sciences and Biopharmaceutics, Guangdong Pharmaceutical University, Guangzhou, Guangdong, China; ^2^ Guangdong Provincial Key Laboratory for Research and Evaluation of Pharmaceutical Preparations, Guangdong Pharmaceutical University, Guangzhou, China

**Keywords:** Schizophyllan, ultrasonic-assisted H_2_O_2_, molecular weight, cell proliferation and migration, tissue regeneration

## Abstract

**Background:**

Schizophyllan (SPG), a bioactive polysaccharide from *Schizophyllum commune*, possesses significant anti-inflammatory, antioxidant, and immunomodulatory properties. The molecular weight of polysaccharides significantly impacts their structural properties and biological functions. However, the functional characteristics of low molecular weight polysaccharides derived from *Schizophyllum commune* remain inadequately explored.

**Methods:**

This study developed an ultrasound-assisted hydrogen peroxide (H_2_O_2_) degradation method to produce low-molecular-weight SPG with enhanced bioactivity. The process was optimized using response surface methodology, focusing on ultrasound duration, ultrasonic power, and H_2_O_2_ concentration. This approach effectively reduced the molecular weight of SPG from 4,409,608 Da to 257,500 Da, yielding three distinct variants: SPG-a (257,500 Da), SPG-b (429,300 Da), and SPG-c (364,800 Da). The bioactivity of these variants was assessed through *in vitro* cell proliferation and migration assays using BJ and HaCaT cells, as well as an *in vivo* zebrafish larval caudal fin regeneration model.

**Results:**

*In vitro*, SPG-b significantly promoted cell proliferation, increasing BJ and HaCaT cells growth by 53.69% and 14.59%, respectively, at a concentration of 300 μg/mL (*p* < 0.05), compared to undegraded SPG. Additionally, scratch assays revealed that SPG-a enhanced BJ cells migration by 24.13% (*p* < 0.05), while SPG-b exhibited most pronounced effect on HaCaT cells migration (17.12%, *p* < 0.05), compared to the undegraded SPG. *In vivo*, SPG-c (3.125 mg/mL) significantly improved fin regeneration rates by 6.97% (*p* < 0.05) in zebrafish larvae, compared to the undegraded SPG.

**Conclusion:**

This study demonstrates that ultrasound-assisted H_2_O_2_ degradation effectively reduces SPG molecular weight while enhancing its functional properties. These findings provide a foundation for the further development of SPG in pharmaceutical and cosmetic applications, highlighting its potential for broader utilization.

## Graphical Abstract

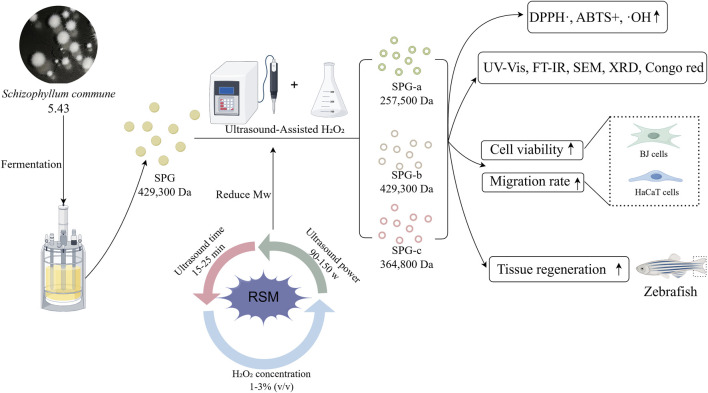

## 1 Introduction

Schizophyllan (SPG), an active polysaccharide originally extracted from the fruiting bodies or fermentation broth of *Schizophyllum commune*, is generally recognized as a glucose-based homopolysaccharide (Zhang et al., 2013). However, several studies have demonstrated that SPG from different sources may exhibit heteropolysaccharide characteristics (Chen et al., 2020; Zhong et al., 2013; Du et al., 2017). Recently, our study (Patent Application No.: 202411835493.0, pending) revealed that SPG produced via liquid fermentation using the *Schizophyllum commune* 5.43 strain (GDMCC No. 5.43), is a heteropolysaccharide consisting of glucose, mannitol, galactose, and fucose in a molar ratio of 86:8:5:1. Notably, SPG obtained through liquid fermentation exhibits potent bioactivities, including anti-inflammatory, antioxidant, and anti-aging effects (Deng et al., 2021; Deng et al., 2022; Tu et al., 2023). Despite these advantages, its high molecular weight and viscosity present significant challenges in pharmaceutical and cosmetic applications. These properties limit its bioavailability by impeding efficient absorption and distribution within the body, while the increased viscosity complicates formulation processes, leading to issues such as poor solubility, instability, and difficulties in product consistency. In contrast, low-molecular-weight polysaccharides offer improved absorption, reduced toxicity, and enhanced bioavailability (Zhao et al., 2016). Several methods have been explored to reduce SPG’s molecular weight. Zhong et al. applied ultrasound-assisted treatment to degrade SPG (Zhong et al., 2015), but this approach is hindered by long processing times, high energy consumption. These findings highlight the critical need to develop efficient and controllable methods for SPG molecular weight reduction to optimize its functionality and broaden its applications.

The biological activity of polysaccharides is closely influenced by their molecular weight, degree of polymerization, and conformation (Wang et al., 2024). Degradation methods for polysaccharides can be broadly categorized into physical, chemical, and biological approaches (Li et al., 2022). While physical methods are environmentally friendly, they are costly and may lead to incomplete degradation (Yu et al., 2020). Chemical approaches are straightforward but risk byproduct generation and environmental hazards (Yuan et al., 2020). Biological methods provide uniform products under mild conditions but are expensive and require stringent controls (Dou et al., 2019). Ultrasonic-assisted degradation has emerged as an eco-friendly, efficient alternative for polysaccharide modification (Hu et al., 2022; Wang et al., 2019; Youssouf et al., 2017). In an ultrasonic-assisted H_2_O_2_ system, ultrasound enhances cavitation effects and accelerates H_2_O_2_ dissociation, producing hydroxyl radicals that promote efficient degradation (Ayanda et al., 2018). This hybrid method combines the advantages of physical and chemical techniques, providing a novel strategy for polysaccharide degradation. However, limited studies have explored this approach specifically for SPG.

Polysaccharides play a critical role in wound healing, which involves vasoconstriction, inflammation, cell proliferation, and tissue remodeling (Pugliese et al., 2018; de Oliveira and Wilson, 2020; Ridiandries et al., 2018; Rodrigues et al., 2019). Fungal polysaccharides, in particular, demonstrate significant potential in promoting tissue regeneration (Sharifi-Rad et al., 2020). For instance, polysaccharides derived from *Trametes versicolor* have been shown to enhance cell proliferation and expedite wound healing (Teymoorian et al., 2024), while extracts from Lignosus rhinocerotis exhibit notable wound repair capabilities (Yap et al., 2023). In comparison, SPG has unique properties in promoting wound healing. Compared with other substances, SPG has good biocompatibility and low toxicity, and it can enhance the body’s immune response to promote tissue repair(Ansari et al., 2025). Despite these promising characteristics, the application of SPG in wound healing and tissue regeneration remains underexplored, particularly regarding the influence of molecular weight variations on its bioactivity.

This study employed ultrasonic-assisted H_2_O_2_ degradation to produce SPG fractions with varying molecular weights. The process parameters, including ultrasonic duration, ultrasonic power, and H_2_O_2_ concentration, were systematically optimized using response surface methodology (RSM). The resulting SPG fractions were evaluated for their structural properties, antioxidant activity, and effects on the proliferation and migration of human skin fibroblasts (BJ) and human keratinocytes (HaCaT). Additionally, their tissue regeneration potential was assessed using a zebrafish caudal fin regeneration model. This research not only elucidates the mechanisms of SPG degradation under ultrasonic-assisted H_2_O_2_ treatment but also highlights the impact of molecular weight on its biological activities, providing a theoretical and technical foundation for the development of low-molecular-weight SPG in pharmaceutical and cosmetic applications.

## 2 Materials and methods

### 2.1 Reagents, materials, and instruments

*Schizophyllum commune* 5.43 (GDMCC No. 5.43), was purchased from Guangdong Microbial Culture Collection Center (Guangzhou, China). 2,2′-Azinobis (3-ethylbenzothiazoline-6-sulfonic) acid Ammonium Salt (ABTS) and 1,1-diphenyl-2-picrylhydrazyl (DPPH) were purchased from Shanghai Macklin Biochemical Technology Co., Ltd. (Shanghai, China). Pullulan polysaccharides were purchased from Showa Denko (Tokyo, Japan). Cyclin B and GAPDH antibodies were purchased from Proteintech (Wuhan, China). CCK-8 and BCA kits were purchased from GLPBIO (Montclair, United States). EdU-594 cell proliferation assay kit was purchased from Beyotime (Shanghai, China). All other chemicals and reagents were of analytical grade and purchased from local suppliers.

The AB zebrafish was provided by Guangzhou Rubi Biotechnology Co., Ltd. HaCaT and BJ cell lines were obtained from Guangdong Marubi Biotechnology Co., Ltd. (Guangzhou, China). High-glucose Dulbecco’s Modified Eagle Medium (DMEM) and trypsin were obtained from Gibco (Grand Island, NY, United States). Fetal bovine serum was obtained from Hangzhou Sijiqing Bioengineering Materials Co., Ltd. (Hangzhou, China).

The following equipment was used: ZQPW-70 shaking incubator with full temperature control, Tianjin Laibote Rui Instrument Equipment Co., Ltd. (Tianjin, China). HC-3026R high-speed refrigerated centrifuge, Anhui Zhongke Zhongjia Scientific Instrument Co., Ltd. (Hefei, China). ELX800 microplate reader, Biotek, (Phoenix, AZ, United States). JY92-LLDN Ultrasound cell disruptor, Ningbo Xinzhi Biotechnology Co., Ltd. (Ningbo, China). NDJ-5S Digital rotational viscometer, Shanghai Genggeng Instrument Equipment Co., Ltd. (Shanghai, China). LC-10A High performance liquid chromatograph, Shimadzu Corporation, (Tokyo, Japan). Thermo Nicoletis 50 Fourier transform infrared spectrometer, ThermoFisher Scientific, (Waltham, MA, United States). JSM-7610F PLUS Field emission scanning electron microscopy, Japan Electronics Co., Ltd. (Tokyo, Japan). D8 ADVANCE X-ray diffractometer, Bruker, (Billerica, MA, United States). Tanon 2,500 Gel imaging system, Tanon Science & Technology Co., Ltd. (Shanghai, China). EVOS M5000 Intelligent Cell Imaging System, Thermo Fisher Scientific (Waltham, MA, United States). SZ680 Continuous Zoom Stereo Microscope, Chongqing Ott Optical Instrument Co., Ltd. (Chongqing, China).

### 2.2 Extraction and preparation of SPG

SPG was extracted from *Schizophyllum commune* 5.43 following our previously established method (Deng et al., 2021). Specifically, *S. commune* 5.43 was cultured in seed medium at 28°C for 3 days to prepare fermentation seed liquid. Subsequently, 5% (v/v) of the seed liquid was inoculated into 250 mL sterile Erlenmeyer flasks containing 100 mL of culture medium. The culture was incubated at 160 rpm and 28°C for 6 days. The medium consisted of 3% glucose, 0.01% KH_2_PO_4_, 0.05% MgSO_4_.7H_2_O, and 0.3% yeast extract, sterilized at 121°C for 20 min. After fermentation, the culture broth was centrifuged at 12,000 g for 20 min to separate the mycelium from the supernatant. The supernatant was collected for further analysis. The total sugar content and reducing sugar contents were determined using the phenol-sulfuric acid method (Yan et al., 2018) and the DNS method (Huang et al., 2020), respectively.

### 2.3 Optimization of the degradation process

#### 2.3.1 Single-factor experimental design

The SPG extracted as described in Section 2.2 was subjected to degradation using an approach modified from Li et al. (2023). Single-factor experiments were conducted to optimize ultrasound duration, ultrasound power, and H_2_O_2_ concentration. For each experiment, the optimized parameter determined from the preceding test was used as a fixed condition. Based on preliminary experiments and insights gained from previous literature (Wang et al., 2024; Li et al., 2020), we have determined the following parameters: Ultrasound durations of 10, 15, 20, 25, and 30 min, ultrasound powers of 50, 100, 150, 200, and 250 W, and H_2_O_2_ concentrations of 1.0%, 2.0%, 3.0%, 4.0%, and 5.0% were evaluated. After each experiment, the kinematic viscosity of SPG samples was measured using a rotary viscometer to determine the effect of each variable. Optimal parameters were identified based on minimum kinematic viscosity.

#### 2.3.2 Evaluation of weight-average molecular weight

The weight-average molecular weight (Mw) of SPG was analyzed using the high-performance liquid chromatography (HPLC) equipped with a refractive index detector and a TSK-Gel G3000 PWXL column, following the method of Gu et al. (2020). SPG solutions prepared in Section 2.2 were filtered through 0.22 µm aqueous microporous membranes before injection. The injection volume was 10 μL, with a flow rate of 0.5 mL/min and a column temperature of 40°C. Pullulan polysaccharides (Mw range: 6.9–883,000 Da) were employed as standards to generate a calibration curve for Mw calculation. This analysis provided an accurate assessment of the molecular weight distribution of SPG after degradation.

#### 2.3.3 Response surface optimization experiment

A Box-Behnken design (BBD) was employed to optimize the degradation process of SPG based on the results of single-factor experiments. This experimental design focused on three key factors: ultrasound power (A), ultrasound duration (B), and H_2_O_2_ concentration (C). Each factor was evaluated at three levels (low, medium, high) to assess its effect on Mw of SPG, which served as the response variable (Rachpirom et al., 2023). The specific levels for each factor are presented in Table 1.

**TABLE 1:** Experimental factors and levels.

Symbol	Factor	Levels		
		−1	0	1
A	ultrasound power (W)	90	120	150
B	ultrasound duration (min)	15	20	25
C	H_2_O_2_ concentration (%, v/v)	1.0	2.0	3.0

#### 2.3.4 Design and analysis

The BBD experimental results were fitted to a quadratic model, and analysis of variance (ANOVA) was conducted to identify the significant factors and interactions influencing Mw. The optimal conditions were determined based on the model predictions and experimental validation.

### 2.4 Structure characterization

#### 2.4.1 Purification of SPG

Activated carbon (1%, w/v) was added to the fermentation broth, followed by incubation in a water bath at 70 °C for 30 min. The mixture was centrifuged at 5,000 g for 30 min to remove pigments. Proteins were removed using the Sevag method (Tang et al., 2024), and polysaccharides were precipitated with ethanol (Wang et al., 2020). The precipitate was dialyzed in pure water for 24 h and lyophilized to obtain purified SPG.

#### 2.4.2 Congo red experiment

The Congo red assay was conducted to examine the interaction of SPG with Congo red as described by Zhang et al. (Zhang et al., 2020). A 0.3 mg/mL SPG solution was mixed with an equal volume of 80 μmol/L Congo red solution. Various final concentrations of NaOH (0–1.0 mol/L) were added to the mixture. A NaOH solution without SPG served as a control. Absorbance spectra between 400 and 600 nm was recorded using a microplate reader, and shifts in the maximum absorption wavelength were analyzed to evaluate conformational changes.

#### 2.4.3 Ultraviolet-visible absorption spectroscopy (UV-Vis) analysis

The UV-Vis spectra of SPG were measured to detect characteristic polysaccharide groups and impurities, following the method of Zhang et al. (2022). SPG solutions (0.3 mg/mL) were scanned within the wavelength range of 200–600 nm using a UV-Vis spectrophotometer, and spectral features indicative of structural integrity and purity were analyzed.

#### 2.4.4 Fourier transform infrared spectroscopy (FT-IR) analysis

Functional groups of SPG were identified using FT-IR spectroscopy. Lyophilized SPG samples were mixed with KBr ground into a fine powder, and compressed into pellets. FT-IR spectra were recorded in the range of 4,000–400 cm^−1^. Peak positions and intensities were analyzed to determine the functional groups and chemical composition of SPG.

#### 2.4.5 Diffraction of x-rays (XRD) analysis

The crystal structure of SPG was analyzed using a D2 PHASER X-ray diffractometer, following the method of Kazemi et al. (2019). Lyophilized SPG samples (3.0 mg) were mounted onto the sample holder and scanned across a 2θ range 5°–90°, a step size of 0.013° and a scanning speed of 12°/min. The diffraction patterns were analyzed to assess crystallinity and structural organization.

#### 2.4.6 Scanning electron microscope (SEM) analysis

The microstructure, particle morphology, and surface characteristics of SPG were visualized using field emission scanning electron microscopy. Lyophilized SPG samples (3.0 mg) were mounted on copper stubs with double-sided conductive adhesive tape, coated with a thin layer of gold, and imaged under the SEM. Surface topography and microstructural features were documented for further analysis.

### 2.5 Determination of antioxidant activity *in vitro*

#### 2.5.1 DPPH· radical scavenging capacity assay

The DPPH radical scavenging activity of SPG was assessed with slight modifications to the method described by Chen et al. (2022). Briefly, 2 mL of the sample was mixed with an equal volume of 0.1 mM DPPH solution and incubated in the dark at room temperature for 30 min. Absolute ethanol was used as the blank control, while ascorbic acid (Vc) served as the positive control. Each sample was analyzed in triplicate. The absorbance of the mixtures was measured at 517 nm using a microplate reader. The DPPH radical scavenging activity was calculated using the following formula:

$$DPPH\cdot \;scavenging\;activity\;\left(\%\right)=\left(1-\frac{A_{1}-A_{2}}{A_{0}}\right)\times 100\%$$

where A_0_ is the absorbance of the DPPH solution in absolute ethanol, A_1_ is the absorbance of the DPPH solution containing the sample, and A_2_ is the absorbance of the sample mixed with absolute ethanol.

#### 2.5.2 ABTS^+^· radical scavenging capacity assay

The ABTS^+^· radical scavenging activity was determined with slight modifications to the method of Zhang et al. (2012). A 7 mM ABTS^+^ stock solution was mixed with 7.35 mM potassium persulfate in a 2:1 (v/v) ratio and incubated in the dark at room temperature for 16 h to generate ABTS^+^· radical. Before use, the solution was diluted with 0.01 M PBS buffer to an absorbance of 0.70 ± 0.02 at 743 nm, serving as the ABTS^+^ working solution. In the assay, 1.9 mL of ABTS^+^ working solution was mixed with 100 μL of the sample and incubated in the dark at room temperature for 7 min. Absorbance was measured at 743 nm using a microplate reader. PBS was used as the blank control, and Vc served as the positive control. The ABTS^+^· radical scavenging activity was calculated using the following formula:

$${ABTS}^{+}\cdot \;scavenging\;activity\;\left(\%\right)=\left(1-\frac{A_{1}-A_{2}}{A_{0}}\right)\times 100\%$$

where A_0_ is the absorbance of the blank control, A_1_ is the absorbance of the ABTS^+^ solution containing the sample, and A_2_ is the absorbance of the solution where the sample was replaced by an equal volume of PBS.

#### 2.5.3 Hydroxyl radical scavenging activity assay

The ⋅OH radical scavenging activity of SPG was determined with slight modifications to the method of Yan et al. (2018) Briefly, 200 μL of the sample was mixed with 600 μL of 6 mM ferrous sulfate solution and 600 μL of 6 mM salicylic acid-ethanol solution. After thorough mixing and incubation at room temperature for 10 min, 600 μL of 8.8 mM hydrogen peroxide solution was added, followed by addition mixing and incubation in the dark for 30 min. The absorbance of the reaction mixture was measured at 517 nm using a microplate reader. Double-distilled water (dd H_2_O) was used as the as the blank control, and Vc served as the positive control. The ⋅OH radical scavenging activity was calculated using the formula:

$$\mathrm{\cdot O}H\;scavenging\;activity\;\left(\%\right)=\left(1-\frac{A_{1}-A_{2}}{A_{0}}\right)\times 100\%$$

where A_0_ is the absorbance of the blank control, A_1_ is the absorbance of the sample, and A_2_ is the absorbance of the reaction mixture in which H_2_O_2_ was replaced by dd H_2_O.

### 2.6 Cell culture

Human immortalized epidermal cells (HaCaT) and human dermal fibroblasts (BJ) were cultured in high-glucose DMEM supplemented with 10% fetal bovine serum (FBS) and 1% penicillin-streptomycin (P/S). Cells were maintained in a humidified incubator at 37°C with 5% CO_2_.

### 2.7 CCK-8 assay

The effect of SPG on cell proliferation was evaluated using the CCK-8 assay, performed according to the manufacturer’s instructions. HaCaT and BJ cells were seeded into 96-well plates at a density of 5.0 × 10^3^ cells per well and incubated for 24 h. Subsequently, the culture medium was replaced with fresh medium containing SPG at various concentrations (0, 50, 100, 200, 300, 400, 500 μg/mL). After 24 h of incubation, the medium was replaced with CCK-8 reagent diluted in medium, and cells incubated at 37°C for 1 h. Absorbance was measured at 450 nm using a microplate reader. Cell viability was calculated to evaluate the proliferative effect of SPG.

### 2.8 Wound healing scratch migration assay

The wound healing assay was conducted to assess the *in vitro* wound-healing potential of SPG, with modifications based on the method described by Li et al. (2022). HaCaT and BJ cells were seeded into 24-well plates at a density of 2.5 × 10^5^ cells per well and cultured until confluent. A uniform scratch was made across the monolayer using a sterile 200 μL pipette tip. Detached cells were removed by gently washing the wells twice with PBS, and fresh medium containing 300 μg/mL SPG was added. The medium without SPG served as the blank control. After 48 h of incubation, images of the wound areas were captured using an optical microscope. The wound healing process was analyzed using ImageJ software, and the cell migration rate was calculated using the following formula:

$$Migration\;rate\;\left(\%\right)=\frac{A_{0}-A_{1}}{A_{0}}\times 100\%$$

where A_0_ represents the initial wound area (pixels) at 0 h, and A_1_ represents the wound area (pixels) at 48 h.

### 2.9 EdU assay

The EdU assay was performed with modifications based on the method described by Zhang et al. (2021) to evaluate cell proliferation. HaCaT cells were seeded into 24-well plates at a density of 1.0 × 10^5^ cells per well and cultured for 24 h. Subsequently, cells were treated with 100 μg/mL SPG for 48 h. To label proliferative cells, an equal volume of 2× EdU working solution was added, followed by incubation at 37°C for 2 h. After incubation, cells were fixed in 4% paraformaldehyde for 15 min and permeabilized with 0.3% Triton X-100 for 5 min. Nuclei were counterstained with DAPI. Fluorescent images of EdU-positive cells were captured using a high-resolution cell imaging system, and the percentage of EdU-positive cells was quantified using ImageJ software.

### 2.10 Western blot assay

Western blotting was conducted to investigate the molecular mechanisms underlying SPG-induced proliferation of HaCaT cells, focusing on Cyclin B1 expression. The method was adapted from An et al. (2024) with modifications. Total proteins were extracted from HaCaT cells treated with 100 μg/mL SPG for 48 h using RIPA lysis buffer supplemented with PMSF. Protein concentrations were quantified using a BCA protein assay kit. Equal amounts of protein (30.0 μg) were separated by 12% SDS-PAGE and transferred to PVDF membranes. The membranes were blocked with 5% non-fat milk at room temperature for 2 h and then incubated overnight at 4°C with primary antibodies against Cyclin B1 (1:5,000) and GAPDH (1:5,000). Following overnight incubation, the membranes were washed and incubated with HRP-conjugated secondary antibodies (1:5,000) for 1 h at room temperature. Protein bands were visualized using a chemiluminescence detection system, and the relative expression of Cyclin B1 was quantified using ImageJ software, with GAPDH serving as the internal control.

### 2.11 Maintenance of larvae zebrafish

Wild-type AB zebrafish were obtained from the Guangzhou Rubi Biotechnology Co., Ltd. and maintained under standardized laboratory conditions (Sun et al., 2019): Key environmental parameters included dissolved oxygen levels >6.0 mg/L, un-ionized ammonia <0.02 mg/L, conductivity at 500–600 μS, nitrate levels <30.0 mg/L, nitrite levels <0.01 mg/L, salinity at 0.6 g/L, and neutral pH. The photoperiod was set to 14 h light:10 h dark, and water temperature was consistently maintained at 28°C. Normally developed embryos were collected following natural spawning and incubated in embryo medium (EM) at 28°C for 72 h. Healthy larvae were selected for subsequent experiments.

### 2.12 Caudal fin regeneration of zebrafish larvae

The caudal fin regeneration assay was performed with slight modifications to the protocol described by Mathew et al. (2006). Zebrafish larvae were anesthetized in 0.05% (w/v) tricaine solution, and caudal fin amputation was carried out under a stereomicroscope. Post-amputation, larvae were immediately transferred to EM containing 3.125 mg/mL SPG and incubated for at 28°C for 72 h. Control groups were maintained in SPG-free EM under identical conditions. At the end of the incubation period, regenerated caudal fins were imaged using a stereomicroscope, and the fin regeneration area was quantified using ImageJ software. The regenerative effects of SPG were assessed by comparing the fin regeneration areas between the SPG-treated and control groups.

### 2.13 Data analysis and statistics

All experiments were conducted in triplicate, and results are expressed as mean ± standard deviation (Mean ± SD). Statistical analyses were performed using one-way ANOVA in SPSS 27.0, with significance determined at *p* < 0.05. Response surface methodology (RSM) analysis was conducted using Design-Expert 13.0. to explore parameter interactions. Graphical representations were generated using GraphPad Prism 10.3 and Origin 2024.

## 3 Results and discussion

### 3.1 Effects of different factors on the kinematic viscosity of fermentation supernatant

Numerous studies have demonstrated that factors such as ultrasound duration, ultrasound power, and H_2_O_2_ concentration significantly influence the molecular weight of polysaccharides (Liu et al., 2024; Li et al., 2019; Cai et al., 2024). Since the kinematic viscosity of polysaccharides correlates closely with their molecular weight (Shao et al., 2020), a single-factor experiment was conducted to evaluate the effects of these variables on the kinematic viscosity of SPG.

**FIGURE 1:**
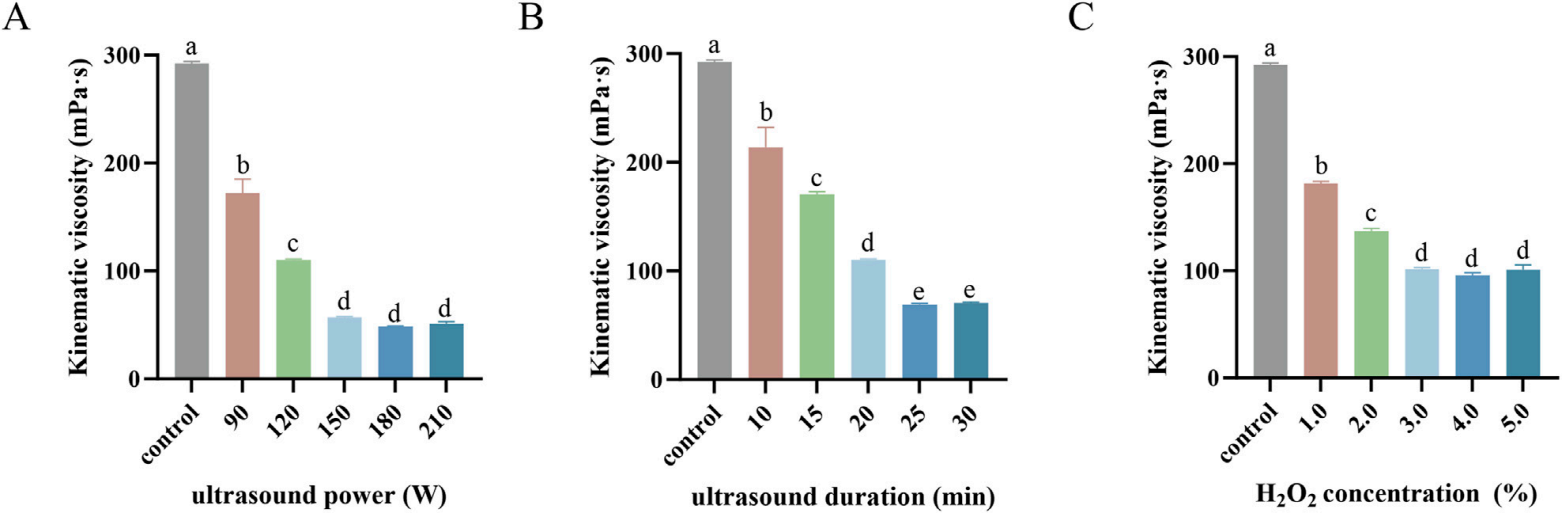
Kinematic viscosity mediated by ultrasound power **(A)**, ultrasound duration **(B)** and H_2_O_2_ concentration **(C)** in single-factor experiments. Values with different letters indicate a significant difference (*p* < 0.05). Data are presented as mean ± SD (N = 3). These findings suggest that each factor contributes to viscosity reduction until saturation, beyond which further increases yield diminishing returns.

As shown in Figure 1A, with the H_2_O_2_ concentration fixed at 2.7% and ultrasound duration set to 20 min, the influence of ultrasonic power on the kinematic viscosity of the fermentation supernatant was analyzed. The kinematic viscosity significantly decreased as the ultrasonic power increased from 0 to 150 W (*p* < 0.05). This decline can be attributed to the intensified cavitation effect at higher ultrasonic power levels (Ogutu and Mu, 2017), which promotes the decomposition of H_2_O_2_ into highly oxidative radicals such as ⋅OH (Ayanda et al., 2018), accelerating SPG degradation. However, when the ultrasound power exceeded 150 W, the viscosity plateaued (*p* > 0.05), likely due to the saturation of the cavitation effect and a dynamic equilibrium in radical concentration, limiting further degradation. Based on these findings, ultrasonic powers of 150 W and 90 W were chosen as the high and low values, respectively, for subsequent response surface analysis.

Figure 1B illustrates the impact of ultrasonic duration on kinematic viscosity with a fixed H_2_O_2_ concentration of 2.7% and ultrasonic power of 100 W. Extending the ultrasonic duration from 0 to 25 min resulted in a significant reduction in the kinematic viscosity (*p* < 0.05), likely due to the cumulative enhancement of the cavitation effect, which increased free radical production and accelerated SPG degradation (Yu et al., 2015; Yan et al., 2016). Beyond 25 min, the viscosity stabilized (*p* > 0.05), possibly reflecting a steady-state molecular weight of SPG molecules due to maximum shear stress and free radical production capacity being reached (Zhou and Ma, 2006). Therefore, ultrasonic durations of 25 min and 15 min were selected as the high and low values, respectively, for response surface analysis.

Figure 1C shows the effect of H_2_O_2_ concentration on kinematic viscosity under fixed conditions of 100 W ultrasonic power and 15 min of ultrasonic duration. Increasing the H_2_O_2_ concentration from 1.0% to 3.0% significantly reduced in kinematic viscosity (*p* < 0.05), likely due to enhanced SPG degradation from increased reactive radical production (Yu et al., 2015). However, beyond 3.0%, no further significant changes were observed (*p* > 0.05), which may be due to excess H_2_O_2_ reacting with ⋅OH radicals to form weaker oxidants such as HO_2_·, or recombination reactions regenerating H_2_O_2_ and reducing free radical availability (Ayanda et al., 2018; Zhou and Ma, 2006). Consequently, H_2_O_2_ concentrations of 3.0% and 1.0% were selected as the high and low values, respectively, for response surface analysis.

### 3.2 Determination of weight-average molecular weight

**FIGURE 2:**
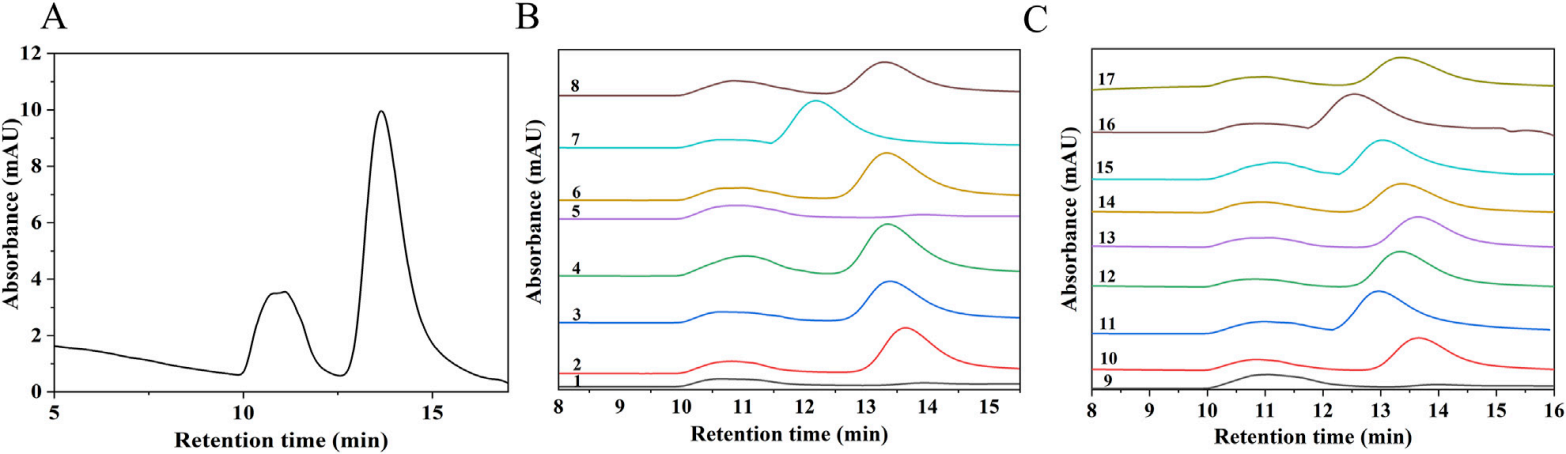
HPGPC chromatograms of SPG before and after degradation. **(A)** Chromatogram of undegraded SPG. **(B, C)** Chromatograms of SPG degradation products. Groups 1–17 represent the experimental conditions established through RSM. The retention times of all 17 groups are significantly reduced after degradation, indicating that the combined application of ultrasound and H_2_O_2_ effectively reduced the Mw of SPG.

To further investigate the effects of ultrasonic power, ultrasonic duration, and H_2_O_2_ concentration on the Mw of SPG, a RSM model was employed. The Mw of both the undegraded SPG and the 17 experimental samples analyzed through RSM was determined using High-Performance Gel Permeation Chromatography (HPGPC), as shown in Figure 2. Figure 2A presents the HPGPC spectrum of the undegraded SPG, with a molecular weight of 4,409,608 Da. Figures 2B, C display the HPGPC spectra for the 17 experimental samples, revealing Mw values ranging from 257,500 to 434,400 Da (detailed values are listed in Table 2). These findings confirm that the combined application of ultrasound and H_2_O_2_ significantly reduced the Mw of SPG, providing valuable experimental data for subsequent investigations.

**TABLE 2:** Box-Behnken experimental design and results.

Run	A-ultrasound power (W)	B-ultrasound duration (min)	C- H_2_O_2_ concentration (%)	Mw (Da)
1	150 (1)	25 (1)	2 (0)	257,500
2	120 (0)	15	3 (1)	424,300
3	120	20 (0)	2	316,500
4	150	20	1 (−1)	304,400
5	90 (−1)	15 (−1)	2	328,200
6	120	25	3	370,100
7	150	15	2	336,000
8	120	20	2	299,800
9	120	20	2	289,200
10	150	20	3	314,500
11	120	15	1	429,300
12	120	25	1	359,900
13	90	20	3	434,400
14	120	20	2	287,300
15	90	25	2	348,700
16	90	20	3	364,800
17	120	20	2	290,300
SPG	—	—	—	4,409,608

### 3.3 Model fitting and optimization

#### 3.3.1 Optimization of SPG’s Mw by RSM

This study employed the Box-Behnken Design (BBD) to optimize the effects of ultrasonic power (A), ultrasonic duration (B), and H_2_O_2_ concentration (C) on the Mw of SPG. The experimental matrix and corresponding data are presented in Table 2. Regression analysis conducted using Design-Expert 13.0 software provided the following fitted regression equation: Y (Mw) = 2.966 × 10^5^ − 32962.50A − 22700.00B + 10612.50C − 24750.00AB − 14875.00AC + 3800.00BC + 10197.50A^2^ + 31177.50B^2^ + 68102.50C^2^.

The statistical parameters of the regression model are summarized in Table 3. The model yielded an F-value of 10.87 (*p* < 0.005), confirming its statistical significance. Additionally, the lack-of-fit test resulted in a p-value of 0.063, exceeding the threshold of 0.05, indicating an insignificant lack of fit. These results validate the model’s suitability for accurately representing the experimental data (Fan and Huang, 2023). The coefficient of determination (R^2^ = 0.9332) and the adjusted R^2^ (0.847) indicate a strong correlation between predicted and observed values, with minimal error. Additionally, the coefficient of variation (C.V.) is 6.11%, well below the acceptable threshold of 10.0%, reinforcing the model’s reliability and accuracy (Han et al., 2016). Regression analysis reveals significant contributions of both linear terms (A and B) and specific quadratic terms (AB, B^2^, and C^2^) to Mw changes (*p* < 0.05). It is noteworthy that the interaction terms AC and BC, along with higher-order coefficients (A^2^), were statistically non-significant (*p* > 0.05). These confirms that ultrasonic power (A) and ultrasonic duration (B) are the primary drivers of SPG degradation (*p* < 0.05). The non-significance of the interaction terms suggests their effects are independent of H_2_O_2_ concentration (C), reinforcing the dominance of ultrasonic parameters.

**TABLE 3:** Variance analysis of regression model results.

Variables	Sum of square	df	Mean square	F-value	*p*-value Prob. > F
Model	4.183 × 10^10^	9	4.648 × 10^9^	10.87	0.0024**
A	8.692 × 10^9^	1	8.692 × 10^9^	20.33	0.0028**
B	4.122 × 10^9^	1	4.122 × 10^9^	9.64	0.0172*
C	9.010 × 10^8^	1	9.010 × 10^8^	2.11	0.1899
AB	2.450 × 10^9^	1	2.450 × 10^9^	5.73	0.0479*
AC	8.851 × 10^8^	1	8.851 × 10^8^	2.07	0.1934
BC	5.776 × 10^7^	1	5.776 × 10^7^	0.1351	0.7241
A^2^	4.378 × 10^8^	1	4.378 × 10^8^	1.02	0.3452
B^2^	4.093 × 10^9^	1	4.093 × 10^9^	9.57	0.0175*
C^2^	1.953 × 10^10^	1	1.953 × 10^10^	45.68	0.0003***
Residual	2.993 × 10^9^	7	4.275 × 10^8^		
Lack of Fit	2.405 × 10^9^	3	8.018 × 10^8^	5.46	0.0673
Pure Error	5.872 × 10^8^	4	1.468 × 10^8^		
Cor Total	4.482 × 10^10^	16			

*R* ^2^ = Coefficients of determination. Significance: *p < 0.05, **p < 0.01 and ***p < 0.001.

#### 3.3.2 Analysis of response surfaces

**FIGURE 3:**
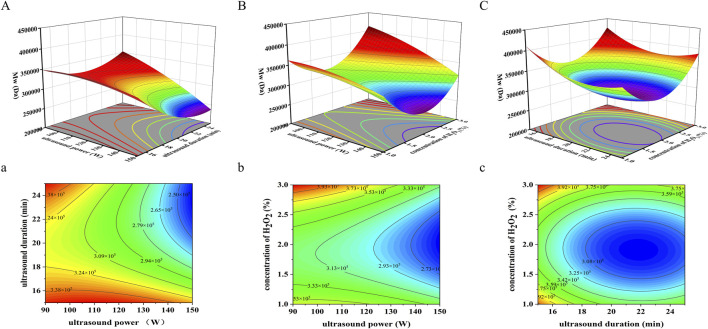
Three-dimensional response surface and contour graphs for the effects on Mw of SPG under the following parameters: **(A)** 3D response surface plot of ultrasound power (W) and ultrasound duration (min). **(a)** 2D contour plot of ultrasound power (W) and ultrasound duration (min). **(B)** 3D response surface plot of ultrasound power (W) and H_2_O_2_ concentration (%). **(b)** 2D contour plot of ultrasound power (W) and H_2_O_2_ concentration (%). **(C)** 3D response surface plot of H_2_O_2_ concentration (%) and ultrasound duration (min). **(c)** 2D contour plot of H_2_O_2_ concentration (%) and ultrasound duration (min). These results underscore that ultrasonic power and duration significantly influence Mw during SPG degradation, whereas H_2_O_2_ concentration plays a less critical role in these interactions.

Three-dimensional response surface plots were generated using Design-Expert 13.0 software to visualize the interactions between variables. Circular contour plots indicate weak interactions, while elliptical contours denote significant interactions (Han et al., 2016; Pyun et al., 2015). In the interaction plot between ultrasonic power and ultrasonic duration (Figure 3A), the response surface exhibits a steep gradient, and the elliptical contour plot (Figure 3a) confirms a significant interaction (*p* = 0.0479 < 0.05). In contrast, the interaction between ultrasonic power and H_2_O_2_ concentration (Figure 3B) is characterized by a more gradual response surface and an elliptical contour plot (Figure 3b), suggesting a non-significant interaction (*p* = 0.1934 > 0.05). Similarly, for ultrasonic duration and H_2_O_2_ concentration (Figure 3C), the response surface is steep, but the circular contour plot (Figure 3c) indicates an insignificant interaction (*p* = 0.7241 > 0.05). These results underscore that ultrasonic power and duration significantly influence Mw during SPG degradation, whereas H_2_O_2_ concentration plays a less critical role in these interactions.

Using response surface analysis, the optimal conditions for achieving an Mw of approximately 360,000 Da were predicted: ultrasonic power of 120 W, ultrasonic duration of 19.06 min, and an H_2_O_2_ concentration of 1.0%. The experimentally obtained Mw of degraded SPG under these conditions was 489,778 Da, approximately 1.36 times higher than the theoretical prediction. This discrepancy may result from several factors. First, SPG’s high molecular weight and broad molecular weight distribution, which complicate uniform degradation during ultrasonic processing (Zhong et al., 2015). Second, the high viscosity of the SPG aqueous solution, which may impede efficient ultrasound transmission, leading to uneven intensity distribution within the reaction medium (Miano et al., 2019). These factors collectively contribute to deviations from the predicted Mw values.

To assess the biological activity of different molecular weights of SPG, three representative Mw samples were selected from the response surface analysis, SPG-a (257,500 Da, Group 1), SPG-b (429,300 Da, Group 11), and SPG-c (364,800 Da, Group 16). The undegraded SPG (SPG-o) served as the control. All samples underwent purification and structural characterization prior to biological activity evaluations.

### 3.4 Structure characterization

#### 3.4.1 Ultraviolet-visible spectroscopy analysis

**FIGURE 4:**
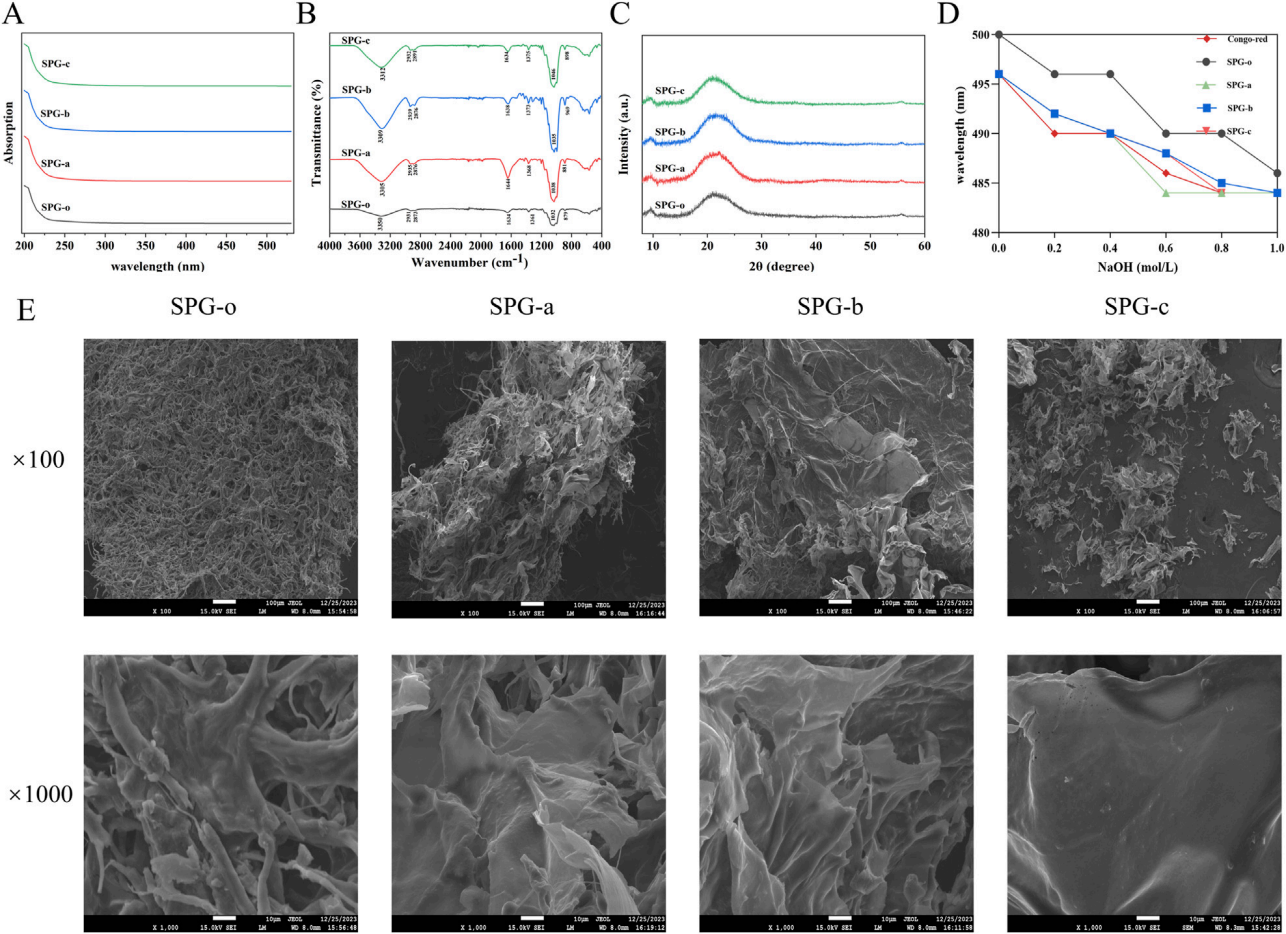
Structural characterization of SPG. **(A)** UV-Vis spectra. The analysis showed that all SPG samples were absent of nucleic acid and protein impurities. **(B)** FT-IR spectra. Revealing characteristic polysaccharide absorption peaks and confirming the preservation of pyranose-type polysaccharides and β-glycosidic bonds after degradation. **(C)** XRD spectra. Indicating SPG’s predominantly amorphous structure, which remains unchanged after ultrasonic-assisted H_2_O_2_ degradation. **(D)** Congo red interaction assay. The triple helical structure of the degraded SPG samples was disrupted. **(E)** SEM images. Showing changes in surface morphology. SPG-o: Undegraded SPG, Mw = 4,409,608 Da. SPG-a: Degraded SPG, Mw = 257,500 Da. SPG-b: Degraded SPG, Mw = 429,300 Da. SPG-c: Degraded SPG, Mw = 364,800 Da.

The UV-Vis spectra of SPG, scanned within the range of 200–600 nm (Figure 4A), exhibited no characteristic absorption peaks at 260 nm and 280 nm. This confirms the absence of nucleic acid or protein impurities in the purified SPG samples, indicating a high level of purity suitable for downstream analyses.

#### 3.4.2 Fourier transform infrared spectroscopy analysis

The FT-IR spectra of SPG-o, SPG-a, SPG-b, and SPG-c (Figure 4B), revealed characteristic absorption peaks typical of polysaccharides. The broad and strong absorption peak at 3350 cm^−1^ corresponds to the O-H stretching vibrations (Li et al., 2023), while the peak near 2,931 cm^−1^ is attributed to C-H stretching vibrations (Wu et al., 2022). The absorption band around 1,634 cm^−1^ is associated with the bound water within the SPG matrix (Gao et al., 2022). Additionally, the peaks within the 1,000–1,200 cm^−1^ range represent the characteristic vibrations of pyranose rings, and the peak near 879 cm^−1^ indicates the presence of β-glycosidic bonds (Ning et al., 2021). These spectral features confirm that SPG primarily comprises pyranose-type polysaccharides linked by β-glycosidic bonds. Significantly, following ultrasonic-assisted H_2_O_2_ degradation, the functional groups in SPG-a, SPG-b, and SPG-c remained unchanged, preserving the pyranose ring structure and glycosidic bond types in the main chain. This indicates that the degradation process did not alter the fundamental chemical composition of SPG.

#### 3.4.3 X-ray diffraction analysis

The crystal structure of SPG was analyzed using X-ray diffraction (XRD) (Figure 4C). The diffraction patterns of SPG-o, SPG-a, SPG-b, and SPG-c exhibited two broad peaks centered at 2θ = 9.7° and 21.7°, characteristic of amorphous materials (Xiao et al., 2022). This confirms that SPG has a predominantly amorphous structure. The ultrasonic-assisted H_2_O_2_ degradation process did not induce significant changes in the crystal structure of SPG. The retention of the amorphous nature suggests that the structural integrity of SPG was largely maintained despite molecular weight reduction.

#### 3.4.4 Congo red interaction assay

The binding characteristics of SPG and its structural integrity were evaluated using Congo Red. Polysaccharides with a triple helical structure exhibit a red shift in maximum absorption wavelength upon binding to Congo Red in alkaline conditions. As sodium hydroxide concentration increases, triple helices disassemble into single helices or random coils, resulting in a blue shift in the maximum absorption wavelength (Guo et al., 2021). The undegraded SPG-o displayed a pronounced red shift under alkaline conditions, indicative of its intact triple helical structure (Figure 4D). In contrast, SPG-a, SPG-b, and SPG-c did not exhibit significant red shifts, suggesting disruption of the triple helical conformation. This disruption can be attributed to the cavitation effects, turbulent shear forces, and free radicals (e.g., ·OH, HO^2^·, H·) generated during the ultrasonic - assisted H_2_O_2_ treatment. These forces likely cleave glycosidic bonds and destabilize the hydrogen bonding network, which is essential for maintaining the triple helical structure (Shi et al., 2022). These findings suggest that ultrasonic-assisted H_2_O_2_ degradation significantly alters the conformational integrity of SPG, providing a mechanistic basis for its modified physicochemical and biological properties.

#### 3.4.5 Scanning electron microscopy analysis

Scanning electron microscopy (SEM) was used to assess microstructural changes in degraded SPG samples (Figure 4E). The fibrous structures characteristic of SPG-o was replaced by plate-like configurations in SPG-a, SPG-b, and SPG-c. Notably, SPG-c exhibited distinct voids and a more flattened, plate-like morphology. These morphological changes are attributed to ultrasonic treatment, where cavitation effects, shock waves, and turbulent shear forces generate localized high-pressure zones, cleaving glycosidic bonds (Shi et al., 2022). Additionally, free radicals generated by H_2_O_2_ decomposition (e.g., ·OH, HO^2^·, and H·) may interact with glycosidic bonds, promoting cleavage and contributing to the observed microstructural changes (Dai et al., 2017). Similar phenomena were reported by Shi et al. (2022) and Zheng et al. (2022), where ultrasonic treatment significantly altered the surface structures of pectin and chitosan, respectively. These findings highlight the critical role of ultrasonic and oxidative processes in modifying polysaccharide microstructures, which may impact their physicochemical and biological properties.

### 3.5 *In vitro* antioxidant activity analysis

**FIGURE 5:**
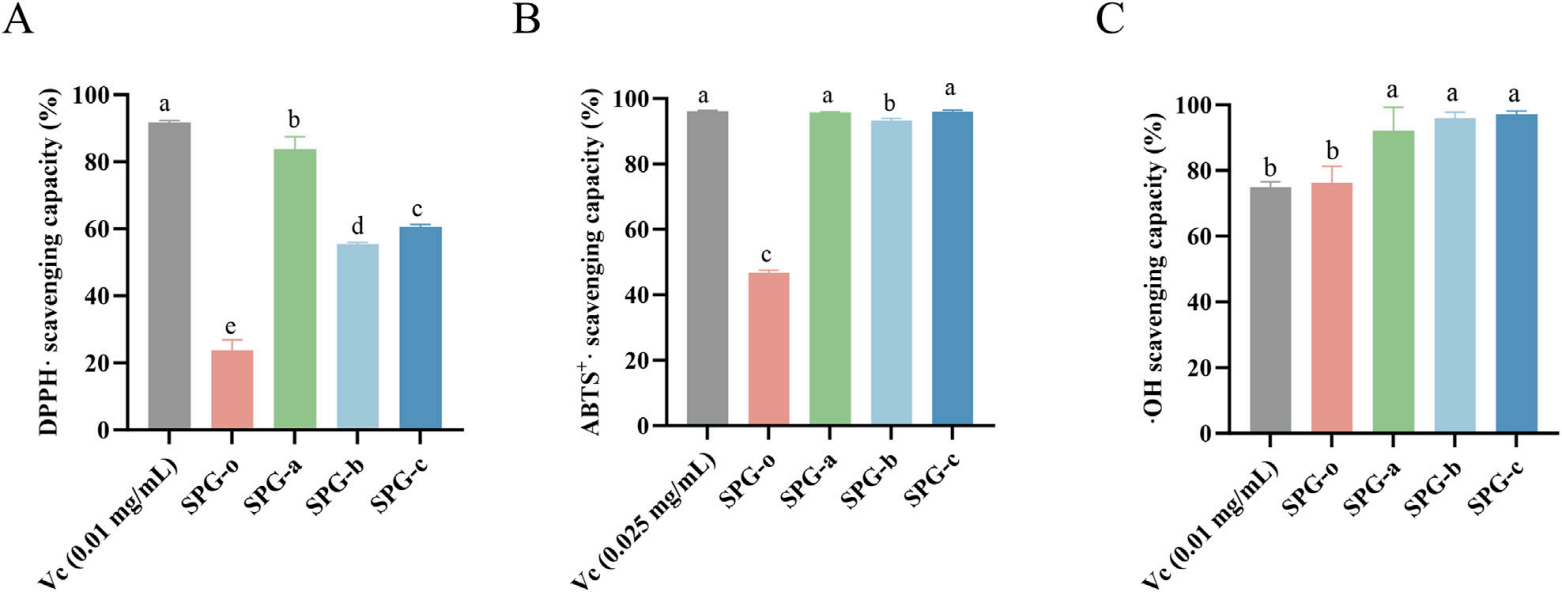
*In vitro* antioxidant activity among different groups, **(A)** DPPH scavenging capacity, **(B)** ABTS^+^· scavenging capacity, and **(C)** ⋅OH scavenging capacity. SPG-o: Undegraded SPG, Mw = 4,409,608 Da. SPG-a: Degraded SPG, Mw = 257,500 Da. SPG-b: Degraded SPG, Mw = 429,300 Da. SPG-c: Degraded SPG, Mw = 364,800 Da. Values with different letters indicate a significant difference (*p* < 0.05). Data are presented as mean ± SD (N = 3). All variants of SPG significantly enhanced the antioxidant capacity against DPPH·, ABTS^+^·, and ⋅OH.

The radical scavenging capacity of degraded SPG variants (SPG-a, SPG-b, SPG-c) was evaluated using DPPH·, ABTS^+^·, and ⋅OH assays, with undegraded SPG-o and Vc as controls (Figure 5). These assays are established methods for assessing the antioxidant activity of polysaccharides. The DPPH radical scavenging assay, valued for its simplicity and reproducibility, showed that SPG-a, SPG-b, and SPG-c exhibited scavenging activities of 83.76% ± 3.68%, 55.43% ± 0.55%, and 60.55% ± 0.77%, respectively, all significantly higher than that of SPG-o (20.37% ± 7.57%, *p* < 0.05) (Figure 5A). Notably, SPG-a demonstrated the highest scavenging activity among the degraded variants (*p* < 0.05). These results indicate that ultrasonic-assisted H_2_O_2_ degradation significantly enhances SPG’s antioxidant, consistent with the findings from Wu et al. (2022).

The ABTS^+^· radical scavenging, a robust indicator of *in vitro* antioxidant capacity (Mirzadeh et al., 2020), revealed scavenging activities of 95.79% ± 0.19%, 93.30% ± 0.67%, and 95.91% ± 0.58% for SPG-a, SPG-b, and SPG-c, respectively (Figure 5B). These values significantly higher than that of SPG-o (46.67% ± 0.85%, *p* < 0.05). Additionally, SPG-a and SPG-c demonstrated significantly greater scavenging activity than SPG-b (*p* < 0.05).

The ⋅OH radical scavenging assay, a critical measure of antioxidant activity due to the high reactivity and biological damage potential of ⋅OH radicals (Kaźmierczak-Barańska et al., 2020), showed that SPG-a, SPG-b, and SPG-c exhibited scavenging activities of 92.00% ± 7.27%, 95.94% ± 1.83%, and 97.01% ± 1.09%, respectively (Figure 5C). These values significantly exceeded that of SPG-o (76.26% ± 5.00%, *p* < 0.05).

In summary, the degraded SPG variants, particularly SPG-a and SPG-c, demonstrated robust free radical scavenging capacities. This enhanced activity is attributed to the disruption of the triple helical structure during ultrasonic-assisted H_2_O_2_ degradation, which exposes a greater number of reactive hydroxyl groups (Yan et al., 2016). These findings suggest that degraded SPG variants hold significant promise as potent free radical scavengers for applications in antioxidative therapies and functional products.

### 3.6 Effect of SPG on wound healing in BJ and HaCaT cells

#### 3.6.1 CCK-8 assay

**FIGURE 6:**
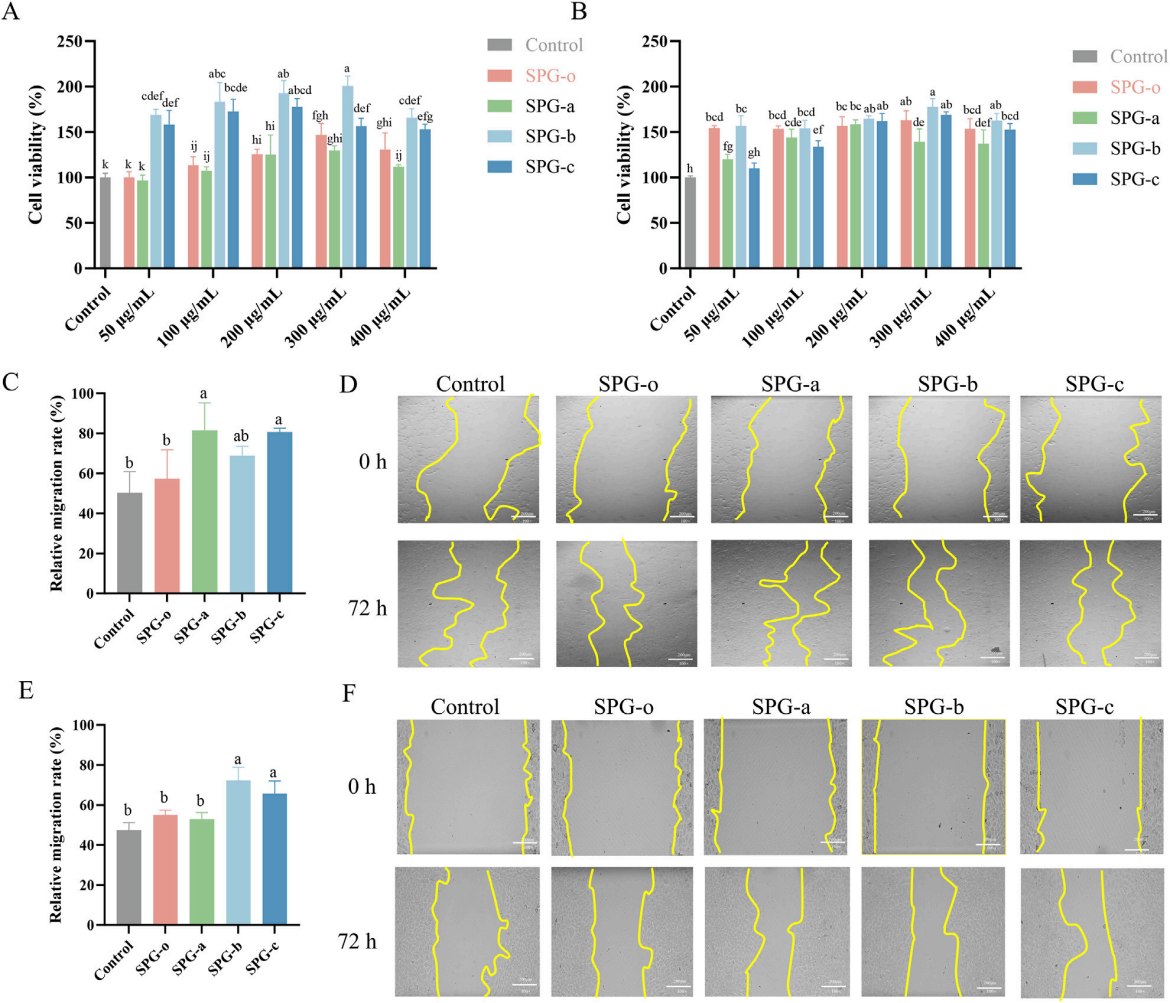
Effects of SPG with different molecular weights on cell viability and migration. Cell viability of BJ **(A)** and HaCaT **(B)** cells after 24 h of treatment. Migration rate of BJ cells after 48 h of treatment **(C)**, with representative images **(D)**. Migration rate of HaCaT cells after 48 h of treatment **(E)**, with representative images **(F)**. Scale bar = 200 μm. Untreated cells (Control) were normalized to 100%. SPG-o: Undegraded SPG, Mw = 4,409,608 Da. SPG-a: Degraded SPG, Mw = 257,500 Da. SPG-b: Degraded SPG, Mw = 429,300 Da. SPG-c: Degraded SPG, Mw = 364,800 Da. Values with different letters indicate a significant difference (*p* < 0.05). Data are presented as mean ± SD (N = 3). SPG-b showed the highest proliferative effect on both BJ and HaCaT cells. Notably, SPG-a most effectively promoted wound healing in fibroblasts, while SPG-b excelled in keratinocyte migration.

Polysaccharide molecular weight significantly influences their biological activities, including cell proliferation modulation. Studies have shown that lower molecular weight polysaccharides, such as fucoidans, exhibit enhanced cytotoxic and modulatory effects on tumor and immune cells (Ummat et al., 2024; Jiang et al., 2022). The effect of SPG-a, SPG-b, and SPG-c on the proliferation of BJ and HaCaT cells was evaluated using the CCK-8 assay. The results demonstrated a concentration-dependent enhancement in cell proliferation across a range of 50–300 μg/mL (Figures 6A, B). Among the variants, SPG-b exhibited the highest proliferative effect at 300 μg/mL, achieving a proliferation rate of 200.42% ± 11.00% in BJ cells and 177.62% ± 9.16% in HaCaT cells (*p* < 0.05). The enhanced proliferative effect of SPG-b (Mw = 429,300 Da) can be attributed to its molecular weight, which strikes an optimal balance between bioavailability and cellular interaction. This intermediate molecular weight likely facilitates efficient cellular uptake and better interaction with cell surface receptors (Pan et al., 2024). These results suggest that lower molecular weight SPG variants, particularly SPG-b, significantly enhance cell proliferation, highlighting their potential applications in promoting cellular biological activity and tissue repair.

#### 3.6.2 Wound healing scratch migration assay

BJ and HaCaT cells play crucial roles in granulation tissue formation and re-epithelialization during wound healing (Zhao et al., 2024; Belvedere et al., 2020). Using a concentration of 300 μg/mL, determined from the CCK-8 assay, the effects of SPG-a, SPG-b, and SPG-c on cellular migration were assessed via a scratch assay. In BJ cells (Figures 6C, D), SPG-o treatment resulted in a wound healing rate of 57.41% ± 14.36%, which did not differ significantly from the control group (50.34% ± 10.51%, *p* > 0.05). In contrast, SPG-a (81.54% ± 13.78%) and SPG-c (80.70% ± 1.92%) significantly enhanced wound healing rates (*p* < 0.05). Similarly, in HaCaT cells (Figures 6E, F), SPG-b (72.24% ± 6.67%) and SPG-c (65.81% ± 6.20%) significantly improved wound healing rates compared to the control group (47.46% ± 3.75%, *p* < 0.05), while SPG-o and SPG-a showed no significant effects (*p* > 0.05). The results indicate that SPG-a exhibited the highest pro-migratory effect on BJ cells, while SPG-b most effectively promoted HaCaT cell migration. This differences may be attributed to cell type-specific interactions with polysaccharides, including variations in receptor expression, uptake efficiency, and downstream signaling activation (Generalov and Yakovenko, 2023).

For BJ cells, SPG-a, with the lowest molecular weight (Mw = 257,500 Da), may be more readily internalized or interact more effectively with fibroblast-associated receptors, such as CD44, which mediates polysaccharide-induced cell migration and adhesion, as observed with hyaluronic acid (Lin et al., 2023). In contrast, SPG-b, with a molecular weight of 429,300 Da, exhibited the strongest effect on HaCaT cell migration. Larger polysaccharides like SPG-b may engage epidermal growth factor receptors (EGFR) more effectively, activating downstream signaling pathways such as phosphoinositide 3-kinase (PI3K)/Akt and mitogen-activated protein kinase (MAPK), which are crucial for keratinocyte proliferation and migration (Zhou et al., 2025).

These findings suggest that the molecular weight of SPG variants differentially influences fibroblast and keratinocyte migration through distinct receptor-mediated mechanisms, highlighting their potential for tailored wound healing applications. However, further studies are needed to elucidate the precise molecular mechanisms involved.

#### 3.6.3 EdU assay

**FIGURE 7:**
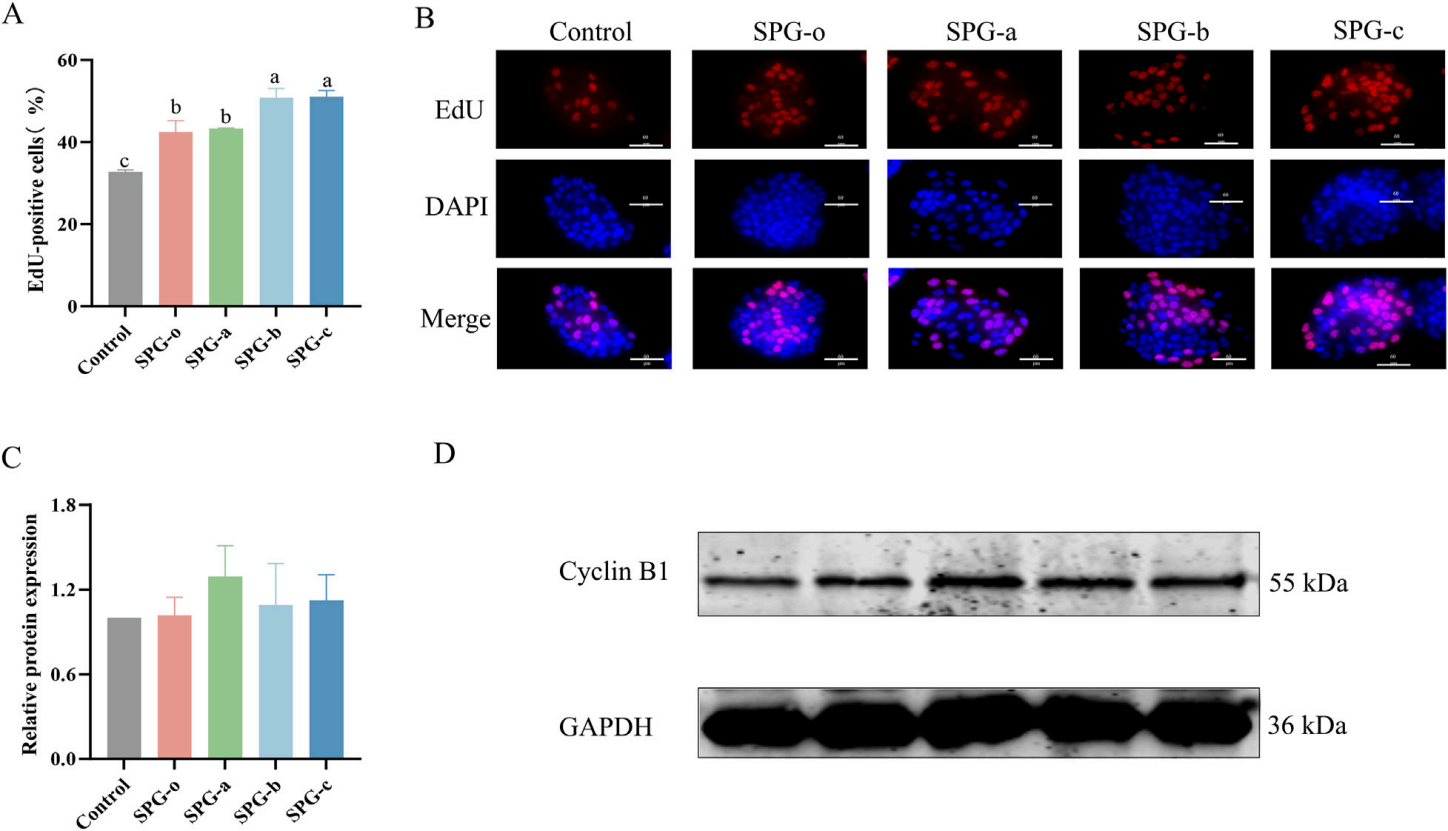
Effects of SPG with different molecular weights on HaCaT cell proliferation and Cyclin B1 expression. Percentage of EdU-positive cells **(A)**. Representative EdU staining images of HaCaT cells **(B)**. EdU - positive are shown in red, DAPI - stained nuclei in blue, and merge is the combination. Scale bar = 60 μm. All SPG variants significantly promoted cell proliferation in HaCaT cells, Quantification of Cyclin B1 expression levels **(C)** and Western blot analysis using anti-Cyclin B1 antibodies **(D)** treated with SPG for 48 h. Response of untreated cells (Control) was considered as 1. Western blot results showed that heightened proliferation did not correlate with major changes in Cyclin B1 levels, implying SPG’s proliferative effect might bypass the G2/M cell cycle phase. SPG-o: Undegraded SPG, Mw = 4,409,608 Da. SPG-a: Degraded SPG, Mw = 257,500 Da. SPG-b: Degraded SPG, Mw = 429,300 Da. SPG-c: Degraded SPG, Mw = 364,800 Da. Values with different letters indicate a significant difference (*p* < 0.05). Data are presented as mean ± SD (N = 3).

EdU staining is a highly sensitive method for detecting cell proliferation by labeling cells during the DNA synthesis (S) phase of the cell cycle (Che et al., 2023). As shown in Figures 7A, B, HaCaT cells treated with SPG variants (SPG-o, SPG-a, SPG-b, and SPG-c) for 72 h exhibited significantly higher proportions of EdU-positive cells compared to the control group (32.71% ± 0.31%, *p* < 0.05). The proportions of EdU-positive cells were 42.42% ± 1.66% for SPG-o, 43.30% ± 0.09% for SPG-a, 50.90% ± 1.26% for SPG-b, and 51.06% ± 0.88% for SPG-c. Notably, the lower molecular weight SPG variants, SPG-b and SPG-c, significantly outperformed SPG-o in promoting EdU incorporation (*p* < 0.05). These findings are consistent with the results from the CCK-8 and scratch assays, further validating the enhanced proliferative and migratory activities of SPG-b and SPG-c in HaCaT cells. The consistency across multiple experimental approaches underscores the potential of SPG-b and SPG-c as effective agents for promoting cell proliferation and wound healing.

#### 3.6.4 Western blot assay

Western blot analysis (Figures 7C, D) revealed that after 48 h of treatment with SPG-a, SPG-b, and SPG-c, Cyclin B1 protein expression in HaCaT cells was slightly elevated compared to the blank control and SPG-o groups. However, these differences were not statistically significant (*p* > 0.05). Cyclin B1 is crucial during the G2/M phase of the cell cycle, and its expression level reflecting the proportion of cells in this phase (Schmitt et al., 2006). The limited effect of SPG treatment on the G2/M phase of HaCaT cells likely accounts for the lack of significant changes in Cyclin B1 expression. This suggests that the primary mechanism by which SPG variants enhance HaCaT cells proliferation may not involve the G2/M phase. Further investigations are necessary to elucidate the molecular pathways underlying the biological effects of SPG.

### 3.7 Effect of SPG on fin regeneration in larvae zebrafish

**FIGURE 8:**
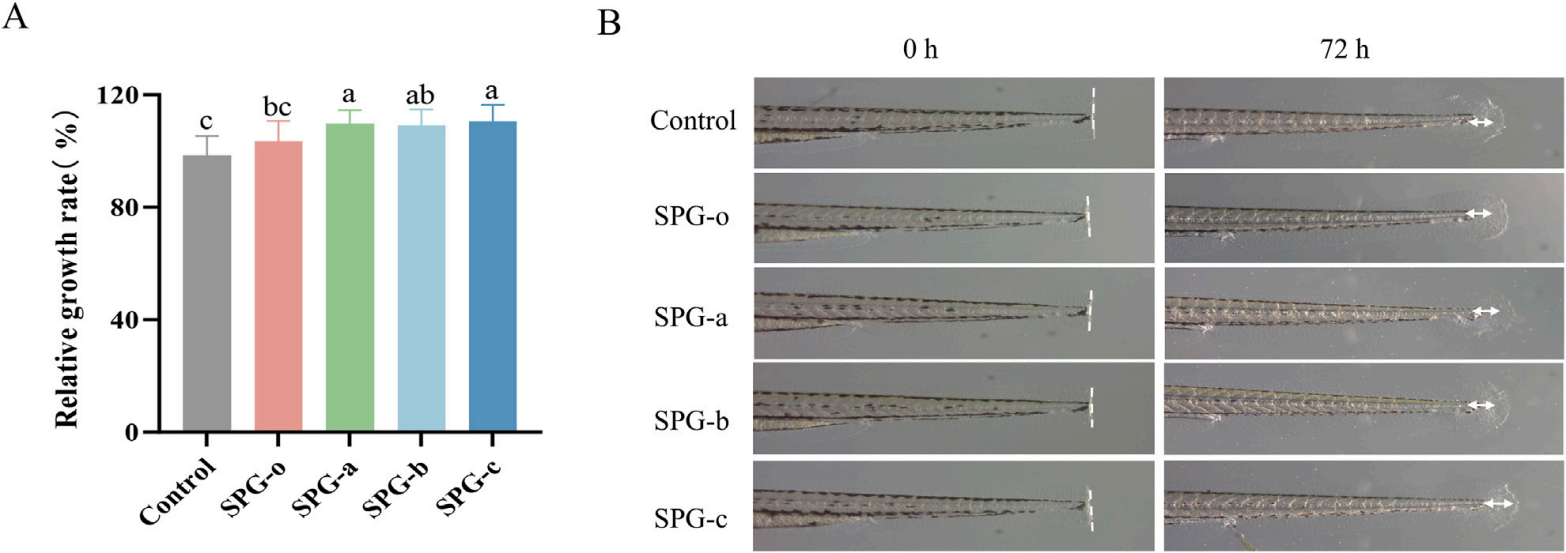
Effect of different molecular weights of SPG exposure on caudal fin regeneration in larval zebrafish. Quantitative analysis of the larval fin regeneration area **(A)**. Representative images showing the larval fin regeneration in group exposed to different molecular weights of SPG (3.125 mg/mL) and the control group **(B)**. SPG-o: Undegraded SPG, Mw = 4,409,608 Da. SPG-a: Degraded SPG, Mw = 257,500 Da. SPG-b: Degraded SPG, Mw = 429,300 Da. SPG-c: Degraded SPG, Mw = 364,800 Da. Values with different letters indicate a significant difference (*p* < 0.05). Data are presented as mean ± SD (N = 15). All variants of SPG significantly enhanced tissue regeneration in the caudal fins of zebrafish.

Zebrafish share approximately 87% genomic homology with humans (Cao et al., 2019), and their remarkable ability to regenerate caudal fins (Gemberling et al., 2013) makes them an excellent model for studying the effects of bioactive compounds on wound healing and tissue regeneration. In this study, we employed the zebrafish caudal fin regeneration model to assess the *in vivo* tissue regenerative potential of SPG variants with different molecular weights. As shown in Figures 8A, B, the fin regeneration rates for SPG-o, SPG-a, SPG-b, and SPG-c were 103.52% ± 7.18%, 109.71% ± 4.81%, 109.04% ± 5.79%, and 110.49% ± 5.94%, respectively, all significantly higher than the control group (98.46% ± 6.91%, *p* < 0.05). Among these, the lower molecular weight variants, SPG-a and SPG-c, exhibited the most pronounced regenerative effects. These results demonstrate that reducing molecular weight significantly enhances the tissue regenerative activity of SPG, highlighting its potential for applications in tissue repair and regeneration. The zebrafish model not only confirms the bioactivity of SPG variants in a physiologically relevant system but also emphasizes the importance of molecular weight modulation in optimizing the therapeutic potential of bioactive polysaccharides. While zebrafish share genetic similarities with humans, key differences in tissue regeneration mechanisms exist. For instance, prolonged TNF-α and MCP-1 elevation post-injury in zebrafish contrasts with rapid resolution in mammals (Smith, 2024). These disparities highlight the need for validation in advanced mammalian models to fully assess SPG’s therapeutic potential.

These findings underline the crucial role of molecular weight reduction in enhancing the tissue regenerative properties of SPG, establishing it as a promising candidate for tissue repair and regeneration applications. Future research should focus on comprehensive structural analyses of the degraded SPG products, including detailed profiling of monosaccharide composition and potential structural modifications, to clarify their correlation with the observed improvements in bioactivity. While the zebrafish model effectively demonstrates the therapeutic potential of low-molecular-weight SPG, the exact mechanisms underlying its tissue repair and wound healing effects remain unclear. In-depth investigations into the associated signaling pathways and gene expression changes will be essential to unravel its mode of action. Additionally, the use of other experimental models and systems will help further validate the therapeutic potential of SPG and support its development for clinical applications in tissue repair and wound healing.

## 4 Conclusion

This study successfully synthesized three molecular weight variants of SPG using an optimized ultrasonic-assisted H_2_O_2_ degradation process. The structural and functional impacts of this method were systematically analyzed. Spectroscopic analyses, including UV-Vis, FT-IR, and XRD, confirmed that while the functional group composition and crystalline structure of SPG remained intact, the triple-helix conformation was disrupted, significantly altering its microstructure. Biological evaluations demonstrated that the low molecular weight SPG exhibited enhanced bioactivity. *In vitro*, the low molecular weight SPG showed significantly improved antioxidant capacity and effectively promoted the proliferation and migration of BJ cells and HaCaT cells, both critical for wound healing. *In vivo*, zebrafish larvae assays confirmed that low molecular weight SPG accelerated caudal fin regeneration, further validating its potential in tissue repair.

In conclusion, molecular weight reduction of SPG significantly enhances its antioxidant and wound healing activities, establishing a solid foundation for its development in cosmetics, pharmaceuticals, and functional foods. This work not only expands the functional scope of SPG but also provides insights into the correlation between molecular weight modulation and the polysaccharide biological. Future research should delve into the specific molecular mechanisms of low molecular weight SPG’s interaction with cellular signaling pathways, as well as its long-term stability and safety for practical application conditions.

## Data Availability

The raw data supporting the conclusions of this article will be made available by the authors without undue reservation.
